# Comparative effectiveness of manipulation, mobilisation and the Activator instrument in treatment of non-specific neck pain: a systematic review

**DOI:** 10.1186/1746-1340-14-7

**Published:** 2006-04-19

**Authors:** Hugh Gemmell, Peter Miller

**Affiliations:** 1 Department of Academic Affairs, Anglo-European College of Chiropractic, Bournemouth, UK

## Abstract

**Background:**

Neck pain is a common problem and different forms of manual therapy are used in its treatment. The purpose of this systematic review was to critically appraise the literature that directly compared manipulation, mobilisation and the Activator instrument for non-specific neck pain.

**Methods:**

Electronic databases (MEDLINE, MANTIS and CINAHL) were searched from their inception to October 2005 for all English language randomised clinical trials that directly compared manipulation, mobilisation and the Activator instrument. Inclusion and exclusion criteria were applied to select the studies and these studies were then evaluated using validated criteria.

**Results:**

Five such studies were identified. The methodological quality was mostly poor. Findings from the studies were mixed and no one therapy was shown to be more effective than the others.

**Conclusion:**

Further high quality research has to be done before a recommendation can be made as to the most effective manual method for non-specific neck pain.

## Background

Neck pain is a common problem [1,2] and manipulation and mobilisation are commonly used by chiropractors, osteopaths and manipulative physiotherapists to treat this condition [3-7]. Numerous surveys [8-12] have shown that the Activator instrument is also widely used within the chiropractic profession for this purpose. Harm from cervical manipulation is unknown, but estimates range from one in 20,000 to five in 10,000,000 [13]. Recent papers [14,15] have suggested that neck pain patients treated with manipulation are more likely to have adverse reactions as compared to those receiving mobilisation. Di Fabio [16], based on a literature review, suggested mobilisation should be used as an alternative to cervical manipulation until more definitive information on the benefits and risks of manipulation is known.

Six systematic reviews [2,13,17-20] have assessed the evidence for the effectiveness of cervical spine manipulation and mobilisation in the treatment of non-specific neck pain, but no systematic review has specifically assessed those studies that directly compared cervical mobilisation, manipulation and the Activator instrument. Is there a difference in effectiveness between manipulation, mobilisation and the Activator instrument in patients with non-specific neck pain?

The purpose of this systematic review was to assess the evidence for the direct comparative effectiveness of manipulation, mobilisation and the Activator instrument on non-specific neck pain.

## Methods

### Inclusion/exclusion criteria

Only randomised clinical trials (RCTs) in the English language were included. RCTs were selected if at least one of the following outcome measures were used: pain level, cervical spine range of motion, pressure pain threshold, global measurement of improvement, and functional status.

Only studies that directly compared cervical manipulation to mobilisation or the Activator instrument were included. The participants recruited had to have non-specific neck pain. Age of participants or duration of symptoms was not considered. Non-specific neck pain was defined as mechanical pain located anywhere between the occiput and upper thoracic spine and the surrounding muscles. Studies including participants with neck pain due to identified pathology were excluded. Studies of participants with headache as the primary disorder were also excluded. Manipulation was defined as high velocity low amplitude thrust to a spinal segment, mobilisation as a low velocity passive or resisted movement within the limit of segmental range of motion, and Activator manipulation as use of a device that delivered a thrust to the spine without causing cavitation.

### Search strategy

The following electronic databases were searched from their inception to October 2005: MEDLINE, MANTIS and CINAHL. The following key words were used: "neck pain", "cervical spine", "manual therapy", "manipulation", "mobilisation/mobilization", "instrument assisted manipulation", and "activator." This initial search strategy was refined using the phrase "cervical manipulation versus mobilisation." These citations were then retrieved and reviewed using the inclusion/exclusion criteria. In addition, the references cited in the papers were then hand-searched for appropriate studies. Each primary author from all the studies was used in another search using MEDLINE to make sure any other appropriate papers were not missed.

### Quality assessment

Two reviewers independently assessed each selected study for quality of methodology, based on the validated five-point Jadad score [21] (Appendix) which ranges from zero to a maximum of five. Where disagreement occurred, the assessment was discussed and consensus reached.

## Results

The initial search strategy identified 217 citations. The refined search yielded 29 citations. Four papers met the inclusion criteria with most studies being excluded because they did not directly compare the interventions of interest. A further paper was identified from examining the references of the 29 papers.

Therefore, five studies involving 489 participants met the relevancy criteria and were included in the quality assessment [22-26]. For a summary of the results see the Table.

The scores for the methodological quality of the studies ranged from zero to three out of a possible five points (Table 1). Most of the studies were of low methodological quality, with the highest quality study [26] scoring three.

Vernon et al. [22] determined the effect of one session of manipulation or mobilisation on pressure pain threshold. Nine participants with mechanical neck pain of less than three months duration were randomised to a manipulation group (n = five) or a mobilisation group (n = four). The mobilisation group received rotational mobilisation with gentle oscillations into the elastic barrier, while the manipulation group received high velocity low amplitude (HVLA) rotational manipulation. The mean improvement in pressure pain threshold for the manipulation group was 45%, while the mean change for the mobilisation group was zero percent. The difference between the groups was significant (P < 0.0001) suggesting manipulation was superior to mobilisation in reducing point tenderness in the tissues surrounding the cervical manipulable lesion.

Cassidy et al. [23] compared the immediate effect of a single manipulation to a single mobilisation in participants with neck pain. One hundred consecutive participants with mechanical neck pain and radiation into the trapezius muscle were randomised to a manipulation group (n = 52) or mobilisation group (n = 48). Outcome measures used were a numerical rating scale (NRS) for pain intensity and goniometric measurement of cervical range of motion. Cervical manipulation consisted of HVLA thrust in rotation away from the painful side. Mobilisation consisted of postisometric relaxation (PIR) type of muscle energy technique to hypertonic muscles restricting joint motion. The mean NRS score decreased 17.3 points in the manipulation group compared to a decrease of 10.5 points in the mobilisation group. The difference between the groups was significant (P = 0.05). Range of cervical motion was improved in both groups; however, the differences were not significant.

Wood et al. [25] compared the effectiveness of diversified HVLA thrusts to the cervical spine with thrusts delivered with an Activator adjusting instrument. Thirty participants with neck pain and restricted cervical range of motion for at least one month were randomised to the two groups with 15 in each group. The leg length analysis as used by Activator Methods was used to determine cervical manipulable lesions for both groups. Each participant was treated until he or she was symptom-free or had received the maximum of eight treatments over four weeks. The Activator group showed a 26% improvement on the Neck Disability Index (NDI), while the manipulation group had a 17% improvement. The difference was not statistically significant. On the NRS the Activator group had a 30% reduction in pain compared to a 17.5% reduction for the manipulation group. This difference was not statistically significant. For the McGill Short-Form Questionnaire the Activator group had a 24.4% improvement with treatment compared to a 26% improvement for the manipulation group. The difference between the groups was not significant. Change in goniometric measurements between the two groups was not significantly different.

Hurwitz et al. [26] compared manipulation to mobilisation in participants with neck pain in a Health Maintenance Organisation (HMO) in the United States. Three hundred thirty six participants were randomised to the following groups: manipulation with and without heat, manipulation with and without electrical stimulation, mobilisation with and without heat, and mobilisation with and without electrical stimulation. Participants were followed for six months with assessments for pain and disability at two and six weeks, and at three and six months. Mobilisation was low velocity variable amplitude movements applied within the participant's passive range of motion. Manipulation was HVLA with minimal extension or rotation. At the two-week follow-up there were no statistically significant differences between mobilisation and manipulation. For most severe pain there was a mean difference of 0.06 on the NRS in favour of manipulation, a difference in average pain of -0.14 in favour of mobilisation and a difference of 1.03 on the NDI in favour of manipulation. At the six-week follow-up there were no statistically significant differences between mobilisation and manipulation. For most severe pain there was a mean difference of 0.4 on the NRS in favour of manipulation, a difference in average pain of 0.23 in favour of manipulation and a difference of 0.92 on the NDI in favour of manipulation. At three-months the differences between manipulation and mobilisation remained non-significant. Most severe pain showed a mean reduction of 0.13 in favour of manipulation. For average pain there was a mean difference of 0.05 in favour of manipulation, for the NDI there was a mean difference of 0.05 in favour of manipulation. At six-months the differences between manipulation and mobilisation remained non-significant. Most severe pain showed a mean reduction of 0.02 in favour of mobilisation. For average pain there was a mean difference of 0.01 in favour of manipulation, for the NDI there was a mean difference of 0.46 in favour of manipulation.

Yurkiw and Mior [24] compared cervical diversified HVLA manipulation to manipulation with an Activator in 14 participants with unilateral mechanical neck pain. Participants were randomised to groups of seven. Outcome measures consisted of the visual analogue scale (VAS) and cervical lateral flexion range of motion determined with a goniometer. Both treatments yielded clinical improvement, but there was no significant difference between the groups. There was a mean difference of 0.037 in favour of the Activator in left lateral flexion and a mean difference of 1.928 in favour of the Activator in right lateral flexion. The mean change in pain between manipulation and Activator was 1.429 in favour of the Activator.

## Discussion

An important result of this review is that very few RCTs exist in this area, and that three of the five studies were pilot in character with investigation limited to a single treatment. Methodological quality was low with weaknesses in trial design noted in small sample size, lack of follow-up, lack of control for placebo response, lack of double blinding, and comparability of relevant baseline characteristics. For these reasons a systematic review was conducted and not a meta-analysis.

Manual therapy treatments are difficult to study in a double-blinded manner and studies assessing the relative effectiveness of different manual therapies may have lower scores because blinding cannot be achieved using current designs. This problem is reflected in this review as none of the studies scored points in the criterion for double-blinding. Sarigiovannis and Hollins [2] suggest that RCTs that do not have a placebo group may be inappropriately penalised, as a placebo in manual therapy that has no effect and is believable by the patient is not available at the present. However, they also state that there is evidence to suggest spinal manual therapy has a significant placebo effect.

This was acknowledged in the current review and the included papers were rescored and points were to be potentially allocated if an adequate time restriction on manual therapy (12 months) was used; however, none of the studies fulfilled this requirement. However, the authors of a recent Cochrane Review [17] suggest that modifying a validated scoring tool to assess RCTs in which double-blinding is not possible is not appropriate. Using a validated instrument to assess methodologic quality, that is not modified, ensures acceptable scientific rigour and quality of the evidence.

Only one paper [26] reported on adverse effects from manual therapy. In any decision on the most appropriate therapy to use for neck pain, not only data on effectiveness are necessary but data on risks are necessary as well. Future studies directly comparing mobilisation, manipulation and instrument assisted manipulation should gather data on adverse effects.

Despite the difficulties involved in RCTs in spinal manual therapy, it is suggested that studies of higher methodological quality are possible. Such studies should include a sham intervention to allow for placebo effects. Detuned ultrasound has been used in some studies of manual therapy [27-31], and although ultrasound is associated with placebo effects, these may be different from manual therapy, mainly due to the lack of a hands-on approach [27]. However, it is suggested that until such time as a true placebo for manual therapy can be developed this type of sham is important to account for placebo effects. Adequate sample size is important. This should be based on sample-size calculation with sufficient power to avoid type I and type II errors. Participant experience with manual therapy and expectation of results from treatment could be offset, to a certain extent, by recruiting participants that have not had manual therapy treatment within the prior 12 months, and by using a questionnaire before inclusion into the study to determine the participant's level of expectation.

The quality of the written report plays an important role in the assessment of methodological quality. If the paper does not report methodological details adequately, a low score may reflect a poorly written report and not on how the study was carried out. However, report writing is a part of the scientific process and it is felt that this is not a weakness of this current review.

A limitation of this review is that only English language papers were included in the literature search. It is possible that an appropriate paper may have been missed if it was published in another language. Another possible limitation is publication bias. No intense effort was made to identify unpublished research. These studies may have had negative outcomes and would be important in any systematic review of treatment effectiveness. However, the authors agree with Bogduk [32] that the databases used were sufficient for finding good quality articles in this area. Greenhalgh and Peacock [33] suggest that citation tracking is an effective search method for locating papers in obscure journals. Using this method we were able to locate one paper that was not identified using the electronic databases.

## Conclusion

Due to the lack in quantity and quality of studies reviewed, more high-quality research needs to be done before a recommendation can be made as to which type of manual therapy has the better effectiveness and safety profile for non-specific neck pain.

## Competing interests

The author(s) declare that they have no competing interests.

## Authors' contributions

HG designed the study, wrote the proposal, performed the literature search, assessed quality of included papers, interpreted results and contributed to writing of the paper. PM assessed quality of included papers, interpreted results and contributed to writing of the paper.

## Appendix

**The Jadad instrument to measure the likelihood of bias in pain research reports **[21]

1. Was the study described as randomised (this includes the use of words such as randomly, random, and randomisation)?

2. Was the study described as double blind?

3. Was there a description of withdrawals and dropouts?

Scoring the items:

Either give a score of 1 point for each "yes" or 0 points for each "no." There are no in-between marks.

Give 1 additional point if: For question 1, the method to generate the sequence of randomisation was described and it was appropriate (table of random numbers, computer generated, etc)

and/or: If for question 2 the method of double blinding was described and it was appropriate (identical placebo, active placebo, dummy, etc)

Deduct 1 point if: For question 1, the method to generate the sequence of randomisation was described and it was inappropriate (participants were allocated alternately, or according to date of birth, hospital number, etc)

and/or: For question 2, the study was described as double blind but the method of blinding was inappropriate (e.g. comparison of tablet vs. injection with no double dummy)

**Table 1:** Summary of Included Trials

**Reference**	Trial Design	**Jadad Score**	**Patients/Problems**	**Interventions**	**Main Outcome Measures**	**Follow-Up Period**	**Main Results**	**Comments**	**Conclusion of Study**
Vernon (1990)^22^	RCT, 2 parallel groups	1	9 with mechanical neck pain	A) HVLA rotational manipulation B) oscillatory mobilisation	Pressure pain threshold	None	Treatment A improved pressure pain threshold more than B	Pilot 1 treatment only	Manipulation superior to mobilisation
Cassidy (1991)^23^	RCT, 2 parallel groups	1	100 with mechanical neck pain and radiation into trapezius	A) HVLA rotational manipulation B) PIR to improve cervical ROM	NRS-101 for pain Cervical ROM	None	Both treatments improved cervical ROM Treatment A superior to B in relieving pain	Pilot 1 treatment only Observer blind	Manipulation superior to mobilisation in relieving pain Both equal in improving cervical ROM
Yurkiw (1996)^24^	RCT, 2 parallel groups	2	14 with unilateral mechanical neck pain	A) diversified HVLA manipulation B) Activator	Cervical lateral flexion VAS for pain	None	Both treatments improved pain and lateral flexion equally	Pilot 1 treatment only Observer blind	No difference between HVLA and Activator
Wood (2001)^25^	RCT, 2 parallel groups	0	30 with mechanical neck pain and restricted ROM	A) diversified HVLA manipulation B) Activator	NRS-101 for pain McGill Neck Disability Index Cervical ROM	1 month	Both treatments had an equal positive effect	Pilot maximum 8 treatments over 4 weeks Non-blinded examiner	No difference between HVLA and Activator
Hurwitz (2002)^26^	2 × 2 × 2 factorial design	3	336 with mechanical neck pain	A) HVLA manipulation with heat B) HVLA manipulation without heat C) HVLA manipulation with EMS D) HVLA manipulation without EMS E) Mobilisation with heat F) Mobilisation without heat G) Mobilisation with EMS H) Mobilisation without EMS	11-point NRS Neck Disability Index SF-36 Adverse reactions Satisfaction with care Patient global assessment	6 months	Both treatments have comparable outcomes Heat and EMS add nothing to treatment	Limited to HMO patients At least 1 treatment Examiner blinded?	Manipulation and mobilisation give comparable clinical outcomes

## References

[B1] Jordan A, Bendix T, Nielsen H, Hansen FR, Host D, Winkel A (1998). Intensive training, physiotherapy, or manipulation for patients with chronic neck pain. Spine.

[B2] Sarigiovannis P, Hollis B (2005). Effectiveness of manual therapy in the treatment of non-specific neck pain: a review. Physical Therapy Review.

[B3] McGregor AH, Wragg P, Gedroyc WMW (2001). Can interventional MRI provide an insight into the mechanics of a posterior-anterior mobilisation?. Clinical Biomechanics.

[B4] Lee RYW, McGregor AH, Bull AMJ, Wragg P (2005). Dynamic response of the cervical spine to posteroanterior mobilisation. Clinical Biomechanics.

[B5] Refshauge KM, Parry S, Shirley D, Larsen D, Rivett DA, Boland R (2002). Professional responsibility in relation to cervical spine manipulation. Australian journal of Physiotherapy.

[B6] Sterling M, Jull G, Wright A (2001). Cervical mobilisation: concurrent effects on pain, sympathetic nervous system activity and motor activity. Manual Therapy.

[B7] Jull G (2002). Use of high and low velocity cervical manipulative therapy procedures by Australian manipulative physiotherapists. Australian Journal of Physiotherapy.

[B8] National Board of Chiropractic Examiners (1993). Job Analysis of Chiropractic: A Project Report, Survey Analysis and Summary of the Practice of Chiropractic within the United States.

[B9] National Board of Chiropractic Examiners (2000). Job Analysis of Chiropractic: A Project Report, Survey Analysis and Summary of the Practice of Chiropractic within the United States.

[B10] National Board of Chiropractic Examiners (2005). Job Analysis of Chiropractic: A Project Report, Survey Analysis, and Summary of the Practice of Chiropractic within the United States.

[B11] National Board of Chiropractic Examiners (1993). Job Analysis of Chiropractic in Canada: A Project Report, Survey Analysis and Summary of the Practice of Chiropractic within Canada.

[B12] National Board of Chiropractic Examiners (1994). Job Analysis of Chiropractic in Australia and New Zealand: A Project Report, Survey Analysis, and Summary of the Practice of Chiropractic within Australia and New Zealand.

[B13] Gross AR, Kay TM, Kennedy C, Gasner D, Hurley L, Yardley K, Hendry L, McLaughlin L (2002). Clinical practice guideline on the use of manipulation and mobilization in the treatment of adults with mechanical neck disorders. Manual Therapy.

[B14] Hurwitz EL, Morgenstern H, Vassilaki M, Chiang L-M (2004). Adverse reactions to chiropractic treatment and their effects on satisfaction and clinical outcomes among patients enrolled in the UCLA neck pain study. J Manipulative Physiol Ther.

[B15] Hurwitz EL, Morgenstern H, Vassilaki M, Chiang L-M (2005). Frequency and clinical predictors of adverse reactions to chiropractic care in the UCLA neck pain study. Spine.

[B16] Di Fabio RP (1999). Manipulation of the cervical spine: risks and benefits. Phys Ther.

[B17] Gross AR, Hoving JL, Haines TA, Goldsmith CH, Kay T, Aker P, Bronfort G (2004). A Cochrane review of manipulation and mobilization for mechanical neck disorders. Spine.

[B18] Hurwitz EL, Aker PD, Adams AH, Meeker WC, Shekelle PG (1996). Manipulation and mobilization the cervical spine. A systematic review of the literature. Spine.

[B19] Koes BW, Assendelft WJJ, Van der Heijden GJMG, Bouter LM, Knipschild PG (1991). Spinal manipulation and mobilisation for back and neck pain: a blinded review. BMJ.

[B20] Bronfort G, Haas M, Evans RL, Bouter LM (2004). Efficacy of spinal manipulation and mobilization for low back pain and neck pain: a systematic review and best evidence synthesis. The Spine Journal.

[B21] Jadad AR, Moore RA, Carroll D, Jenkinson C, Reynolds JM, Gavaghan DJ, McQuy HJ (1996). Assessing the quality of reports of randomised clinical trials: Is blinding necessary?. Controlled Clin Trials.

[B22] Vernon HT, Aker P, Burns S, Viljakaanen S, Short L (1990). Pressure pain threshold evaluation of the effect of spinal manipulation in the treatment of chronic neck pain: A pilot study. J Manipulative Physiol Ther.

[B23] Cassidy JD, Lopes AA, Yong-Hing K (1992). The immediate effect of manipulation versus mobilization on pain and range of motion in the cervical spine: A randomized controlled trial. J Manipulative Physiol Ther.

[B24] Yurkiw D, Mior S (1996). Comparison of two chiropractic techniques on pain and lateral flexion in neck pain patients: a pilot study. Chiropractic Technique.

[B25] Wood TG, Colloca CJ, Mathews R (2001). A pilot randomized clinical trial on the relative effect of instrumental (MFMA) versus manual (HVLA) manipulation in the treatment of cervical spine dysfunction. J Manipulative Physiol Ther.

[B26] Hurwitz EL, Morgenstern H, Harber P, Kominski GF, Yu F, Adams AH (2002). A randomized trial of chiropractic manipulation and mobilization for patients with neck pain: Clinical outcomes from the UCLA neck-pain study. Am J Public Health.

[B27] Ernst E, Harkness E (2001). Spinal manipulation: a systematic review of sham-controlled, double-blind, randomized clinical trials. J Pain Symptom Manage.

[B28] Downing DS, Weinstein A (1986). Ultrasound therapy for subacromial bursitis. A double blind trial. Phys Ther.

[B29] Nykanen M (1995). Pulsed ultrasound treatment of the painful shoulder. A randomized, double-blind, placebo-controlled trial. Scan J Rehabil Med.

[B30] Van der Windt DAWM, Van der Heijden GJMG, Van den Berg SGM, Ter Riet G, De Winter AF, Bouter LM (2002). Therapeutic ultrasound for acute ankle sprains. The Cochrane Database of Systematic Reviews.

[B31] Hashish I, Harvey W, Harris M (1986). Anti-inflammatory effects of ultrasound therapy: evidence for a major placebo effect. Rheumatology.

[B32] Bogduk N (2003). Spinal manipulation for neck pain does not work. The Journal of Pain.

[B33] Greenhalgh T, Peacock R (2005). Effectiveness and efficiency of search methods in systematic reviews of complex evidence: audit of primarysources. BMJ.

